# Basic characterization of antibodies targeting receptors of the tumor necrosis factor receptor superfamily

**DOI:** 10.3389/fimmu.2023.1115667

**Published:** 2023-03-27

**Authors:** Olena Zaitseva, Mohamed Anany, Harald Wajant, Isabell Lang

**Affiliations:** ^1^ Division of Molecular Internal Medicine, Department of Internal Medicine II, University Hospital Würzburg, Würzburg, Germany; ^2^ Department of Microbial Biotechnology, Institute of Biotechnology, National Research Center, Giza, Egypt

**Keywords:** affinity, agonism, antibody, FcγR, *Gaussia princeps* luciferase (GpL), immunotherapy, TNF receptor superfamily

## Abstract

Many new immunotherapeutic approaches aim on the stimulatory targeting of receptors of the tumor necrosis factor (TNF) receptor superfamily (TNFRSF) using antibodies with intrinsic or conditional agonism. There is an initial need to characterize corresponding TNFRSF receptor (TNFR)-targeting antibodies with respect to affinity, ligand binding, receptor activation and the epitope recognized. Here, we report a collection of simple and matched protocols enabling the detailed investigation of these aspects by help of *Gaussia princeps* luciferase (GpL) fusion proteins and analysis of interleukin-8 (IL8) production as an easily measurable readout of TNFR activation. In a first step, the antibodies and antibody variants of interest are transiently expressed in human embryonal kidney 293 cells, either in non-modified form or as fusion proteins with GpL as a reporter domain. The supernatants containing the antibody-GpL fusion proteins can then be used without further purification in cell-free and/or cellular binding studies to determine affinity. Similarly, binding studies with mutated TNFR variants enable the characterization of the antibody binding site within the TNFR ectodomain. Furthermore, in cellular binding studies with GpL fusion proteins of soluble TNFL molecules, the ability of the non-modified antibody variants to interfere with TNFL-TNFR interaction can be analyzed. Last but not least, we describe a protocol to determine the intrinsic and the Fc gamma receptor (FcγR)-dependent agonism of anti-TNFR antibodies which exploits i) the capability of TNFRs to trigger IL8 production in tumor cell lines lacking expression of FcγRs and ii) vector- and FcγR-transfected cells, which produce no or only very low amounts of human IL8. The presented protocols only require standard molecular biological equipment, eukaryotic cell culture and plate readers for the quantification of luminescent and colorimetric signals.

## 1 Introduction

The receptors of the tumor necrosis factor (TNF) receptor superfamily (TNFRSF) are of crucial relevance in a variety of immunoregulatory processes but also control tissue homeostasis and development (1, 2). With respect to cancer immunotherapy there is particular interest in targeting the immunoregulatory TNFRs 4-1BB, cluster of differentiation 27 (CD27), CD40, OX40 and TNF receptor 2 (TNFR2) (3–5). However, also the broadly proinflammatory TNFRs fibroblast growth factor inducible-14 (Fn14) and TNFR1 as well as the cytotoxic TNF-related apoptosis-inducing ligand (TRAIL) death receptors and CD95 attract considerable interest as targets in tumor therapy (6–9).

TNFRSF receptors (TNFRs) become typically activated by binding of transmembranous trimeric ligands of the TNF superfamily (TNFSF). Membrane TNFSF ligands (TNFLs) recruit three TNFR molecules. The resulting complexes of a memTNFL trimer and three TNFR molecules then assemble spontaneously to signaling active clusters due to their high local concentration in the cell-to-cell contact zone between the TNFR- and memTNFL-expressing cells (10). Of overwhelming translational relevance is that FcγR-bound anti-TNFR immunoglobulin G (IgG) antibodies similarly activate TNFRs as memTNFLs (11, 12). In this case, TNFR dimers recruited to FcγR-bound anti-TNFR antibodies, instead of memTNFL-bound TNFR trimers, undergo “activating” clustering in the cell-to-cell contact zone between TNFR- and FcγR-expressing cells. There are also soluble trimeric TNFL molecules originating from the transmembranous form by proteolytic processing or from alternative splicing. Worth mentioning, TNFRs can be categorized according to their responsiveness to soluble ligand trimers and free IgG antibodies. TNFRs of category I spontaneously cluster and strongly signal upon binding of soluble TNFL trimers and become frequently also activated by free, thus not FcγR-bound, IgG1 antibodies. In contrast, category II TNFRs largely fail to cluster and signal despite high-affinity binding of ligand molecules or antibodies (10).

There is good preclinical proof for the antitumoral activity of antibodies targeting the aforementioned TNFRs. There is, however, also growing evidence from preclinical and clinical studies that off-tumor activity causes dose-limiting toxicity (8, 11, 13, 14). Accordingly, there are also rapidly growing efforts to develop novel formats for TNFR-specific antibodies and antibody fusion proteins with conditional or intrinsic FcγR-independent agonism or bifunctionality (8, 11, 13, 14). The dose-limiting toxicity of anti-TNFR antibodies are often related to engagement of FcγR effector functions and/or complement activation but can also be caused by systemic activation of the targeted TNFR itself or the interplay with endogenous ligand molecules. Indeed, the cellular mode of action(s) of both the antitumoral effects and the dose-limiting toxicity of an anti-TNFR antibody *in vivo* are often not fully clear. Several antibody features may play a role here, in particular in the case of category II TNFRs. For example, the subclass of an IgG antibody determines which type of FcγR can serve as a target to constitute agonistic activity of anti-category II TNFRs and eventually controls where in the body and to which extent the targeted TNFR but also antibody effector activities become activated. The epitope recognized by the antibody determines its possible impact on ligand binding of the targeted TNFR. Blocking antibodies as free molecules may act as inhibitors but act as agonists when bound to FcγRs. Non-blocking antibodies could be neutral with respect to TNFL-TNFR interaction but can also be able to synergistically trigger TNFR activation in concert with soluble ligand molecules. Examples for this mode of action are the anti-TNFR2 antibody 80M2 (15), the CD40-specific antibodies S2C6, SGN-14 and CDX-1140 (16–18) or the anti-murine CD95 antibody Jo2 (19). Therefore, the basis for the development of an antibody or antibody fusion protein efficiently stimulating the TNFR of interest without causing unwanted side effects is its thorough characterization with respect to affinity, ligand binding, receptor activation and the epitope recognized.

Here, we summarize a number of methods and protocols on the example of various antibody formats targeting different TNFRs (**Table 1**) that allow to test these parameters quickly and accurately using the basic equipment of molecular and cellular biology laboratory without the need of protein purification, post-translational protein modification or primary immune cells.

**Table 1:** Domain architectures and properties of the recombinant proteins used in this work.

Protein	Description/Domain architecture	Properties
anti-CD40(G28.5)-IgG1	Rec. anti-CD40 IgG1 Ab clone G28.5	CD40-spec., FcγR binding competent, FcγR-dependent agonism
anti-CD40(G28.5)-IgG1-LC : GpL	Rec. anti-CD40 IgG1 Ab clone G28.5 with GpL domain on C-terminus of LC	CD40-spec., FcγR binding competent, luminescent reporter domain, FcγR-dependent agonism
Anti-CD40(ADC)-IgG1	Rec. anti-CD40 IgG1 Ab clone ADC1013	CD40-spec., FcγR binding competent, FcγR-dependent agonism, blocking antibody
Anti-CD40(CP-8…)-IgG1	Rec. anti-CD40 IgG1 Ab clone CP-870/983	CD40-spec., FcγR binding competent, FcγR-dependent agonism, non-blocking antibody
anti-TNFR2(68/69)-IgG1(N297A)	Rec. anti-TNFR2 IgG1(N297A) Ab clone SBT-002 (or 68/69)	TNFR2-spec., strongly reduced FcγR binding, blocking antibody
GpL-TNC-CD40L	GpL domain fused to the N-terminus of the tenascin C trimerization domain followed by the extracellular domain of CD40L	CD40 binding, luminescent reporter domain
TNFR2(ed)-GpL	TNFR2 ectodomain followed by GpL	Luminescent
TNFR2(ed)-CRD1-4-GpL	CRD1-CRD4 of TNFR2 ectodomain followed by GpL	Luminescent
TNFR2(ed)-CRD1-3-GpL	CRD1-CRD3 of TNFR2 ectodomain followed by GpL	Luminescent
TNFR2(ed)-CRD1-2-GpL	CRD1-CRD2 of TNFR2 ectodomain followed by GpL	Luminescent
TNFR2(ed)-CRD1-GpL	CRD1 of TNFR2 ectodomain followed by GpL	Luminescent
3xVHH(V12t)-Fc(DANA)	Three copies of CD40-spec. VHH fused to N-terminus of DANA-mutated human IgG1 Fc	CD40-spec., strongly reduced FcγR binding, intrinsic agonism, blocking antibody
3xVHH(4H04)-Fc(DANA)	Three copies of 41BB-spec. VHH fused to N-terminus of DANA-mutated human IgG1 Fc	41BB-spec., strongly reduced FcγR binding, intrinsic agonism
VHH(V12t)-Fc	CD40-spec. VHH fused to N-terminus of human IgG1 Fc	CD40-spec., FcγR binding competent, FcγR-dependent agonism; blocking antibody
VHH(4H04)-Fc-GpL	41BB-spec. VHH fused to N-terminus of human IgG1 Fc followed by GpL	41BB-spec., FcγR binding competent, luminescent reporter domain, FcγR-dependent agonism, blocking antibody
VHH(1D10V1)-Fc-GpL	OX40-spec. VHH fused to N-terminus of human IgG1 Fc followed by GpL	OX40-spec., FcγR binding competent, FcγR-dependent agonism, luminescent reporter domain
VHH(hzC06)-Fc-GpL	GITR-spec. VHH fused to N-terminus of human IgG1 Fc followed by GpL	GITR-spec., FcγR binding competent, luminescent reporter domain, moderate intrinsic agonism, FcγR-enhanced agonism
3xVHH(V12t)-Fc-GpL	Three copies of CD40-spec. VHH fused to N-terminus of human IgG1 Fc followed by GpL	CD40-spec., FcγR binding competent, luminescent reporter domain, intrinsic agonism, blocking antibody
3xVHH(hzC06)-Fc-GpL	Three copies of GITR-spec. VHH fused to N-terminus of human IgG1 Fc followed by GpL	GITR-spec., FcγR binding competent, luminescent reporter domain, intrinsic agonism

For sequences and references to the source of sequences, please see supplemental data.

## 2 Equilibrium binding studies with anti-TNFR antibody GpL fusion proteins: Determination of affinity and epitope characterization

**Figure 1:**
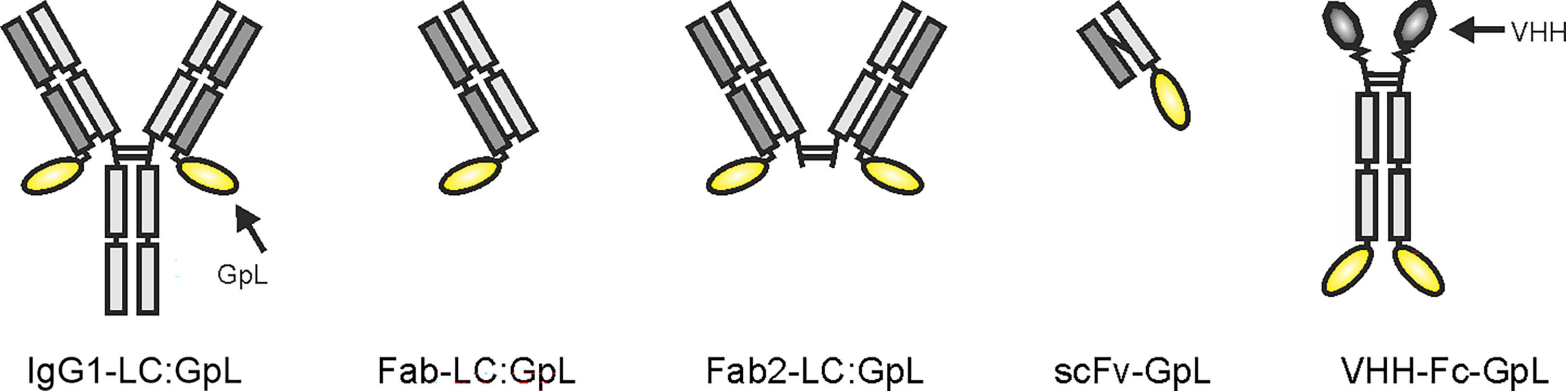
Domain architectures of anti-TNFR antibody GpL fusion proteins useful for the method collection presented.

Starting point of this method and the methods described below is the recombinant availability of the antibody or antibody variant of interest along with a *Gaussia princeps* luciferase (GpL) fusion protein derived thereof. The latter can be easily obtained by genetic fusion of human codon usage-optimized “leader”-less GpL to the C-terminus of the heavy or light chain of an antibody (IgG) or antibody domain (fragment antigen binding (Fab), Fab_2_, fragment crystallizable (Fc), single-chain variable fragment (scFv), variable heavy chain-only antibody (VHH) ( (20–22); **Figure 1**). There is no need to have the GpL antibody fusion protein (GpL-Ab-Fp) available in purified form. Cell culture supernatants (SNs) of transiently transfected eukaryotic cells (e.g. HEK293 or Chinese hamster ovary (CHO) cells) expressing the GpL antibody fusion protein are fully sufficient. Dependent on the structure/tags of the GpL-Ab-Fp, its concentration in the supernatant (SN) can be determined by various means, e.g. using an commercially available IgG ELISA kit, comparison of luciferase activity with a purified GpL fusion protein of known concentration or comparing by western blotting the band intensities of the GpL fusion protein with those of the purified GpL-free protein as standard. Cellular binding studies are then readily possible.

**Figure 2:**
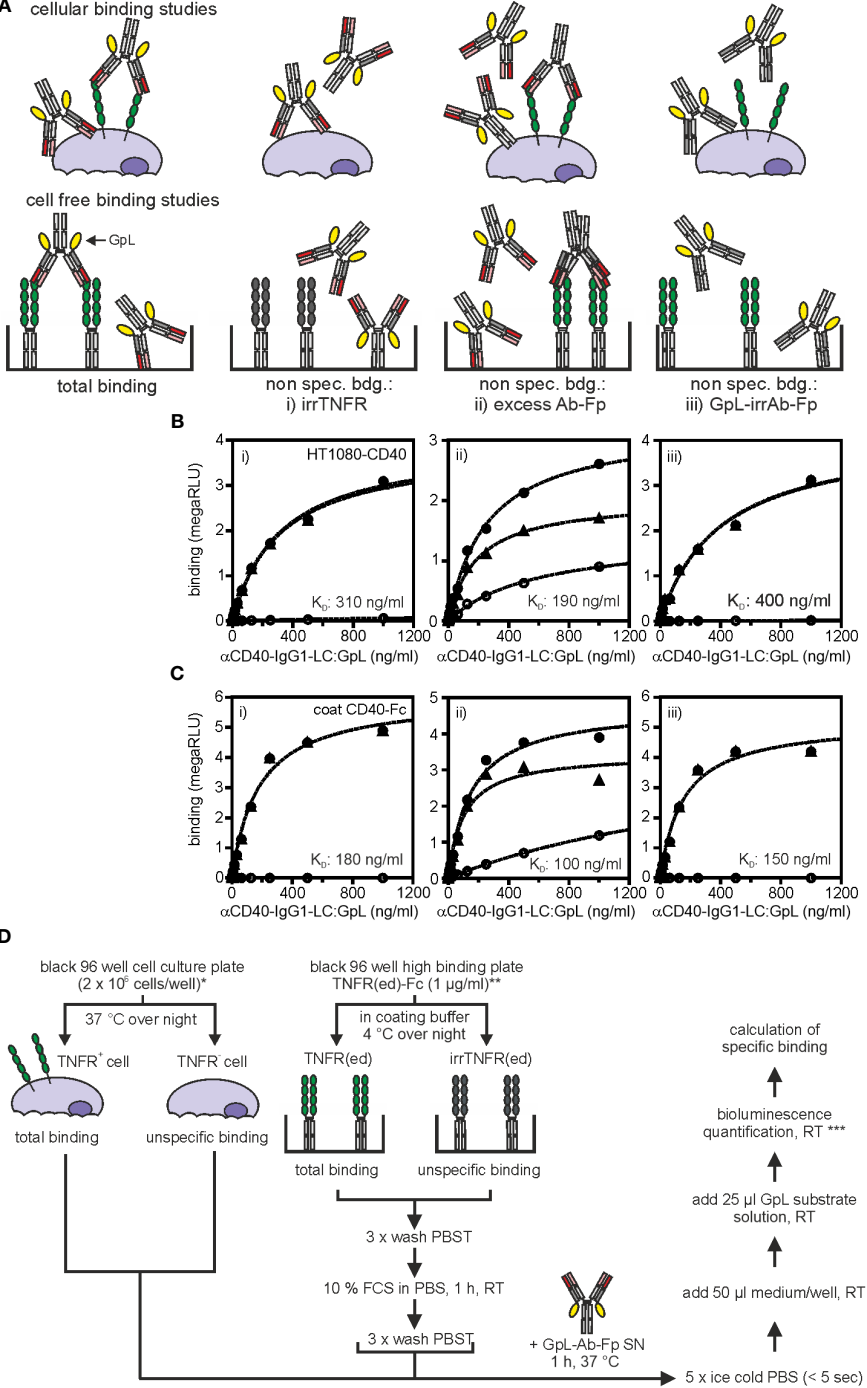
Equilibrium binding studies with anti-TNFR antibody GpL fusion proteins. **(A)** Possible strategies to determine non-specific binding. Upper panel: cellular binding studies; lower panel cell-free binding studies. For further details see main text. **(B)** HT1080-CD40 or HT1080 cells were seeded in black clear bottom cell culture 96 well plates (2 x 10^4^ cells/well) overnight. Next day, specific binding (black triangles) of anti-CD40(G28.5)-IgG1-LC : GpL was determined by subtraction of unspecific binding values (open circles) of either i) target negative HT1080 cells, ii) HT1080-CD40 cells blocked with anti-CD40(G28.5)-IgG1, or iii) HT1080-CD40 cells treated with an antibody GpL fusion protein of irrelevant specificity (irrIgG1-LC : GpL) from the corresponding total binding values (black circles) of anti-CD40(G28.5)-IgG1-LC : GpL to HT1080-CD40 cells. The non-linear regression analysis to a single binding site type of interaction function of the GraphPad Prism 5 software was used to fit the specific binding values. The left three panels show in each case the binding data of one of three independent experiments and the right diagram shows the affinities calculated from the three independent experiments for each of the methods i) to iii) used for determination of non-specific binding values. ANOVA was used to compare affinities. n.s., not significant. **(C)** For cell-free binding studies black 96 well high binding plates were coated with 1 µg/ml of a Fc fusion protein of the extracellular CD40 domain (CD40(ed)-Fc) or a corresponding Fc fusion protein of the extracellular domain of an irrelevant TNFR (irrTNFR(ed)-Fc) in coating buffer at 4°C overnight. Next day, specific binding (black triangles) of anti-CD40(G28.5)-IgG1-LC : GpL was calculated by subtracting the unspecific binding values (open circles) of i) wells coated with irrTNFR(ed)-Fc, ii) wells coated with CD40(ed)-Fc and blocked with an excess of anti-CD40(G28.5)-IgG, or iii) wells coated with CD40(ed)-Fc and incubated with an irrIgG1-LC : GpL fusion protein from the corresponding total binding values (black circles) derived of anti-CD40(G28.5)-IgG1-LC : GpL incubated CD40(ed)-Fc wells. K_D_ values were again calculated *via* by fitting, using the “one site specific binding” function of the GraphPad Prism 5 software. The three left panels again show the binding data of one of three independent experiments and the right diagram shows again the affinities calculated from the three independent experiments for each of the methods i) to iii) used for determination of non-specific binding values. ANOVA was used to compare affinities. n.s. not significant. **(D)** Detailed protocol of cellular (left panel) and cell-free (right panel) equilibrium binding studies with anti-TNFR GpL fusion proteins (Ab-GpL-Fp). * Please note: It is also possible to calculate the receptor numbers per cell with this set up. For this purpose one has to include a well for determination of the cell number per well and must determine the specific luminescence activity per GpL molecule by help of the luminescence of a GpL standard of known concentration. For an example see Fick et al., 2011 (23). ** Purified Fc fusion proteins of the TNFR ectodomain (TNFR(ed)-Fc) can be directly be coated. TNFR(ed)-Fc from cell culture supernatants of cells producing the protein can be used when the wells were pre-coated with protein G. *** To minimize errors due to the inactivation of GpL activity after adding substrate, measure within 20 sec. If this is not possible, ensure that the same time period lies between addition of substrate and recording of luminescence.

Total binding values are obtained by incubation of TNFR^+^ cells (endogenous TNFR expression, TNFR transfectant) with the GpL-Ab-Fp SN, thorough removal of unbound molecules by 5 rapid washes with phosphate-buffered saline (PBS) or medium and measuring of cell-associated luciferase activity (**Figures 2A, B**). Ideally, non-specific binding values are derived of similarly processed TNFR^-^ cells (TNFR knockout cell variant, empty vector (EV) transfectant). Alternatively, non-specific binding values can be obtained from the TNFR^+^ cells when coincubated with a constant excess (> 200 fold of highest GpL-Ab-Fp concentration) of the GpL-free antibody version as competitor (**Figure 2A**). Non-specific binding can also be determined from TNFR^+^ cells treated with a structurally similar GpL-Ab-Fp with irrelevant antigen specificity (**Figure 2A**). Specific binding values are calculated by subtraction of non-specific binding values from the corresponding total binding values. According to our experience, the non-specific binding values obtained by competition with an excess of the conventional antibody are sometimes higher than those obtained with cells lacking antigen expression or using an irrelevant GpL antibody fusion protein (e.g. **Figures 2B, C**). This is expected and plausible since even when a high excess of the competitor molecule can be reached, what is often challenging with respect to the amount and concentration of the competitor required, there remains unavoidable a minor component of specific binding in the non-specific binding values. Nevertheless, the trending “lower” quality of the non-specific binding values obtained by competition does usually not result in a statistical significant difference in the finally calculated affinity (**Figures 2B, C**).

Due to the excellent brightness of GpL and its over many orders of magnitude linear activity (24), 10.000-100.000 cells per data point (=cells/well) are typically sufficient to yield robust specific binding values for antibody variants with a dissociation constant (K_D_)-value of 25 nM or better and cells expressing > 1000 receptors/cell. The choice of the number of cells used for generation of a data point is eventually mainly dependent from the type of cell used in the experiment. For binding studies with adherent cells, a 96-well plate format is convenient, in which, dependent on the size of the cells, 10.000 or 20.000 cells per well were seeded the day before the experiment. In the case of the use of suspension cells, we recommend to increase the number of cells to 100.000 or even 1.000.000 per sample point, not necessarily to improve sensitivity but rather to ensure that the cell pellet is easily visible after centrifugation to facilitate the washing steps. For notoriously low expressed TNFRs, e.g. TNFR1, we recommend the use of transiently expressed TNFR mutants lacking the intracellular domain. Affinity can also be determined in cell-free assays when the TNFR ectodomain is available in recombinant form enabling direct (purified) or indirect immobilization (SN with tagged protein) to an ELISA plate (**Figures 2A, C**). Exemplary cellular and cell-free equilibrium binding studies performed this way are shown in **Figures 2B, C** for a GpL-Ab-Fp of the CD40-specific antibody G28.5 (25). The general process of this type of binding studies has been summarized in **Figure 2D**.

**Figure 3:**
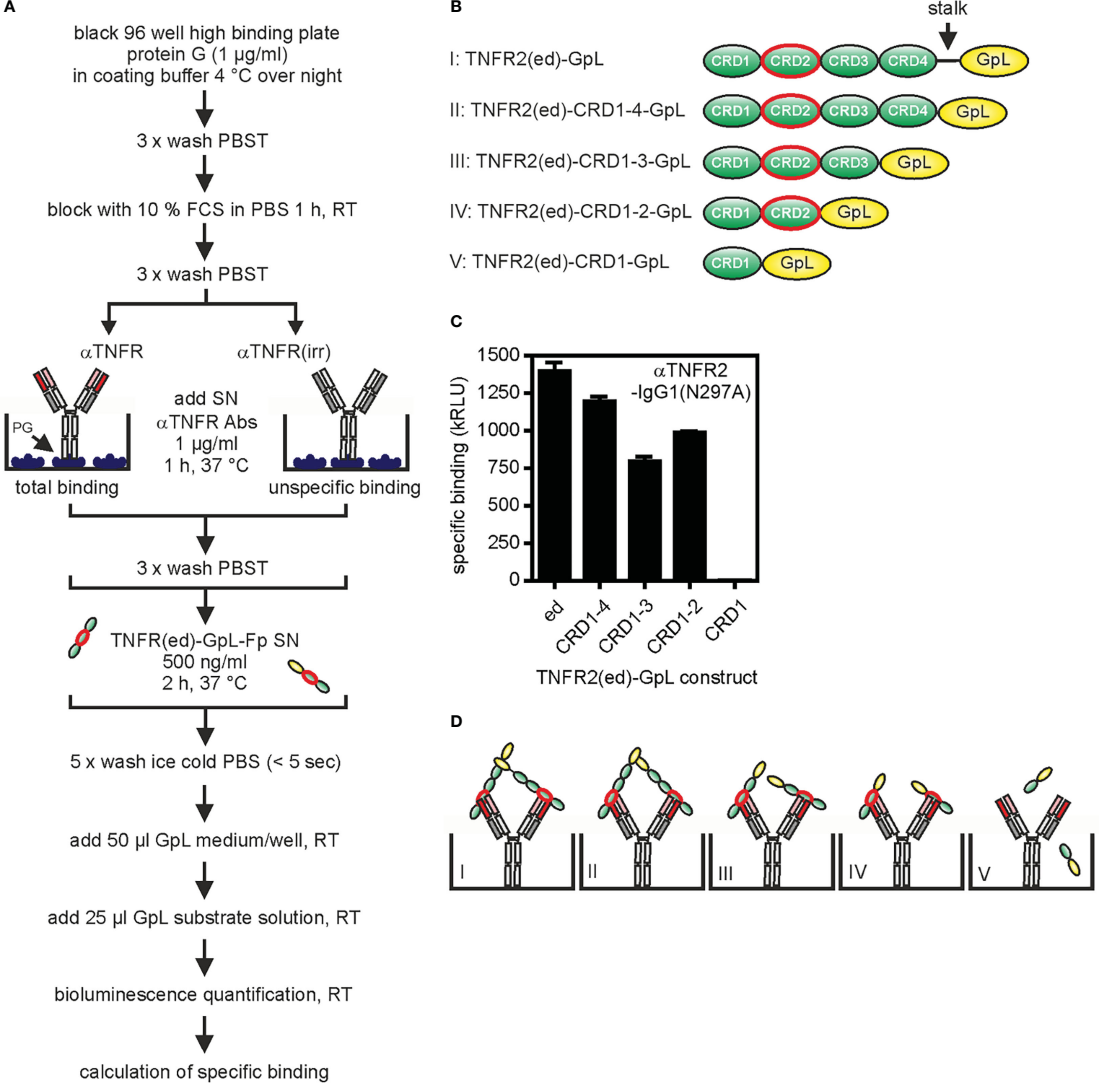
Epitope mapping for the anti-TNFR2 antibody 68/69 using TNFR-GpL fusion proteins. **(A)** Process chart of the method. **(B)** Schemes of the domain architecture of TNFR2(ed)-GpL fusion proteins used. The cysteine rich domain 2 (CRD2) of TNFR2 is circled in red. **(C)** Black 96 well high binding plates were coated with 1 µg/ml protein G (PG) in coating buffer at 4°C overnight. Next day, anti-TNFR2(68/69)-IgG1(N297A) or an irrelevant IgG1 were bound to the immobilized protein G by incubation for 1 h at 37°C. After removal of unbound proteins, 500 ng/ml of the various TNFR2-GpL fusion proteins were added (2 h, 37°C). Finally, unbound molecules were removed and well-associated luminescence was measured. Specific binding of the TNFR2 deletion mutant molecules was obtained by subtraction of the unspecific binding values (irr. IgG1 wells) from the total binding values (anti-TNFR2(68/69)-IgG1(N297A) wells) of the relevant TNFR2(ed)-GpL fusion proteins. GpL medium: RPMI 1640 medium supplemented with 0,5% fetal calf serum (FCS); GpL substrate solution: 1,5 µM coelenterazine in PBS. **(D)** Scheme of the interaction of the various TNFR2-GpL fusion proteins with PG-immobilized anti-TNFR2 antibody.

The methodology described above for the determination of antibody affinity can be identically applied to TNFR mutants (deletion mutants, point mutants, receptor chimeras) and TNFR homologues from other species. This way, it is possible to pinpoint/characterize the epitope recognized of the GpL-Ab-Fp and its antigen species specificity. A complementary approach to map the recognized epitope is to capture the antibody (without a GpL domain) of interest on an ELISA plate and qualitatively evaluate binding of GpL fusion proteins with wild-type or mutated parts of the ectodomain (**Figure 3A**). For example, GpL-tagged deletion mutants of the TNFR2 ectodomain lacking the stalk region, the stalk region + cysteine-rich domain 4 (CRD4) or the stalk region + CRDs 3 and 4 all efficiently bind to the anti-TNFR2 antibody 68/69 immobilized on plastic (**Figures 3B, C**). In contrast, a GpL fusion protein of the TNFR2 CRD1 alone showed no binding at all (**Figures 3B, C**). These data suggest that this anti-TNFR2 antibody interacts with the CRD2 of TNFR2 (**Figure 3D**).

## 3 Effect of anti-TNFR antibodies on ligand-receptor interactions

**Figure 4:**
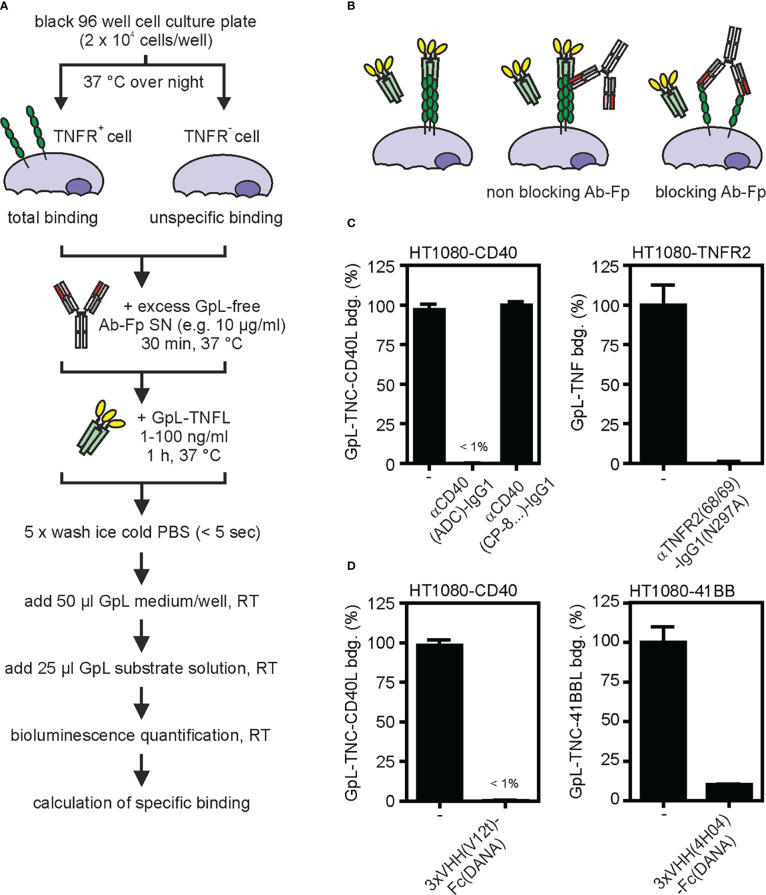
Effect of anti-TNFR antibodies on TNF ligand binding. **(A)** Process chart of the method. **(B)** Scheme of ligand binding of blocking or non-blocking TNFR antibody fusion proteins (Ab-Fp). **(C, D)** The indicated HT1080-transfectants and corresponding TNFR negative HT1080 cells were seeded in black clear bottomed 96 well cell culture plates overnight (2 x 10^4^). Next day, cells were incubated with an excess of the indicated “GpL-free” anti-TNFR antibody (10 µg/ml) **(C)** or “GpL-free” single domain antibody (VHH) Fc fusion proteins **(D)** for 30 minutes. Then a TNFR-specific GpL-TNFL was added in a concentration close to its K_D_ value (typically 1 – 100 ng/ml) and remaining specific binding was determined.

For the understanding of potential *in vivo* effects of anti-TNFR antibodies, it is of crucial relevance to know the impact of the anti-TNFR antibody or antibody fusion protein on ligand binding. With very few exceptions, TNFRs interact with ligands of the TNF superfamily. TNF superfamily ligands (TNFLs) are typically homotrimeric proteins and occur in membrane-bound and soluble form (26). Importantly, TNFRs can differ in their response to binding of soluble TNFLs. While one category of TNFRs (e.g. TNFR1, death receptor 3 (DR3), GITR) become efficiently activated by binding of soluble and membrane-bound ligand trimers, a second category of TNFRs (e.g. TNFR2, CD27, CD40, CD95, 41BB, OX40) efficiently signals in response to membrane-bound TNFLs but is not or only poorly activated by binding of soluble TNFL molecules (10). With respect to TNFL binding anti-TNFR antibody variants can have quite different effects. Antibody variants can completely prevent TNFL binding or can have no effect on TNFL binding at all, and some antibodies can even enhance binding of TNFLs at low concentrations. The impact of anti-TNFR antibodies on ligand-receptor interaction can be straightforwardly addressed by competition binding studies with anti-TNFR antibodies or antibody fusion proteins and GpL-TNFL fusion proteins (**Figure 4A**).

In a comprehensive previous study, we found that an N-terminal GpL domain does not or only poorly interfere with the dose response activity of conventional and oligomerized variants of soluble TNFLs suggesting that the GpL-TNFL fusion proteins bind in a similar fashion to TNFRs as conventional TNFL molecules (27). To figure out only qualitatively whether an anti-TNFR antibody interferes with TNFL-TNFR interaction, it is sufficient to simply check whether a huge excess of the antibody molecule affects receptor binding of the TNFR-specific GpL-TNFL fusion protein in cellular binding assays (**Figures 4B–D**). For example, an variant of the anti-CD40 antibody ADC-1013 (28) completely blocked the binding of soluble GpL-TNC-CD40L to CD40-expressing cells while a corresponding variant of the anti-CD40 antibody CP-870/983 (29) showed no effect in the same setting (**Figure 4C**). Similar competition assays with a IgG1(N297A) variant of the antagonistic TNFR2-specific antibody SBT-002 (30), here designated as 68/69, revealed > 99% inhibition of binding of GpL-TNF (**Figure 4C**). Using this type of assay, we further demonstrated strong inhibitory effects of the hexavalent Fc fusion protein variants 3xVHH(V12t)-Fc(DANA) and 3xVHH(4H04)-Fc(DANA) of the CD40- and 41BB-specific nanobodies V12t (31) and 4H04 (32) on binding to CD40- and 41BB-expressing cells (**Figure 4D**). If the affinity of the GpL-TNFL fusion protein for the TNFR of interest is known, one can also determine the inhibitor constant of the antibody in heterologous competition binding studies. The TNFR affinity of the GpL-TNFL fusion proteins, which is required for the latter purpose, can easily be determined using the protocols described above for antibody GpL fusion proteins (**Figure 2**).

## 4 Effect of FcγR binding on the agonistic activity of anti-TNFR antibodies

Early on, it has been recognized that the binding to FcγRs can strongly enhance the receptor-stimulating ability of anti-TNFR antibodies. There is now overwhelming evidence that antibodies targeting category II TNFRs are often poorly agonistic as free molecules but regularly gain high agonistic activity when presented to TNFRs in FcγR-bound form (11, 12, 33). Similarly, anti-category II TNFR antibodies equipped with cell surface antigen-recognizing scFv domains also acquire strong agonism when anchored *via* this scFv domain to the targeted antigen (e.g (34).), thus when presented in an membrane-attached manner resembling membrane-bound TNFLs or FcγR-bound anti-TNFRs. The agonism-conferring effect of FcγR binding can even convert antagonistic into agonistic antibodies. Comprehensive knowledge and assessment about the intrinsic agonism of an anti-TNFR antibody in free and FcγR-bound form can be gained by coculture assays of TNFR responder cells without endogenous FcγR expression and FcγR-transfected cells. The use of the latter instead of cells with endogenous FcγRs enables the evaluation of the effect of a single type of FcγR and also results in high expression levels which ensure as good as possible that each TNFR molecule can be indeed occupied by a FcγR-bound antibody molecule. Since all signaling competent members of the TNFRSF stimulate the classical NFκB pathway, upregulation of NFκB-regulated factors are useful to quantify TNFR engagement. The expression of the chemokine IL8 is dominantly induced *via* the classical NFκB pathway (35) and its TNFR-induced upregulation has been frequently reported for various cell lines. Since IL8 production can be easily quantified by ELISA, we use it in this protocol as a read-out for TNFR activation. Of course, other simply evaluable read-outs for TNFR activation can be used as well, provided it can be ensured that the “read-out” measured exclusively or dominantly originates from the TNFR responder cell population.

**Figure 5:**
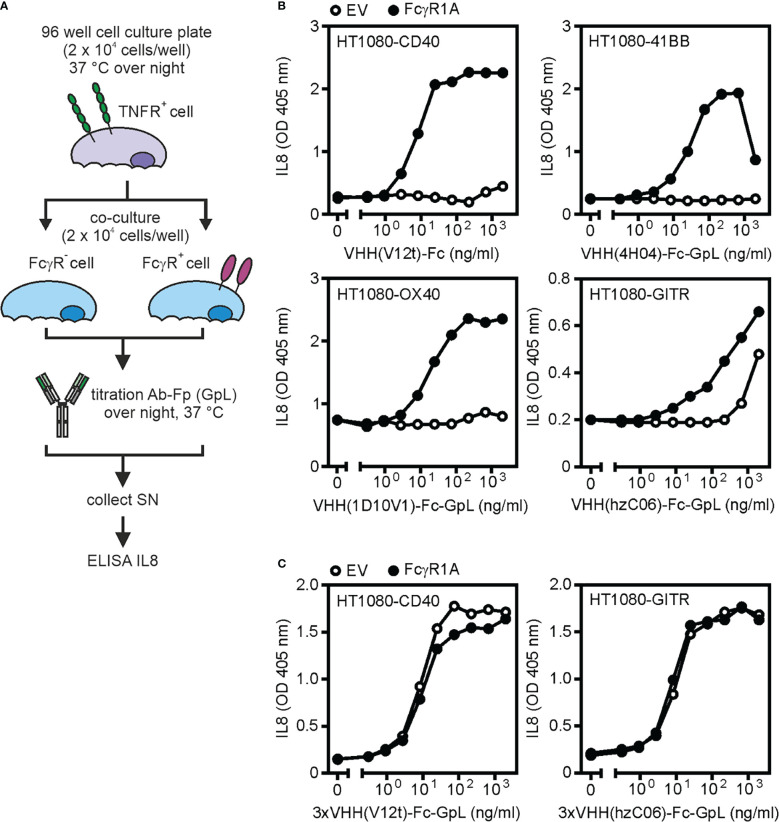
FcγR-dependent and -independent activity of antibodies targeting TNFRs. **(A)** Process chart of method. **(B, C)** The indicated HT1080-transfectants have been treated as indicated with the TNFR-specific VHH-Fc **(B)** or VHH(3x)-Fc **(C)** fusion proteins VHH(V12t)-Fc (CD40-specific), VHH(4H04)-Fc-GpL (41BB-specific), VHH(1D10V1)-Fc-GpL (OX40-specific), VHH(hzC06)-Fc-GpL (GITR-specific); 3xVHH(V12t)-Fc-GpL (CD40-specific) and 3xVHH(hzC06)-Fc-GpL (GITR-specific) along with HEK293 cells transfected with empty vector (EV) or an expression plasmid encoding FcγR1A. Next day, the amount of IL8 in the cell culture supernatants was determined. Upregulation of IL8 production served as the readout for TNFR activation. SN: supernatant.

To quantify the potential impact of FcγR binding for the agonistic activity of anti-TNFR antibodies the dose response relationships of IL8 induction by an anti-TNFR antibody are side by side determined for cocultures of appropriate TNFR responder cells with FcγR transfectants and corresponding empty vector (EV) control transfectants (**Figures 5A–C**). Cocultures of TNFR responder cells and transfectants expressing the membrane-bound form of the ligand of the investigated TNFR can serve as a benchmark if needed (21). One day before stimulation the responder cells are seeded in a 96 well plate and efficiently transfectable cells, producing no or low amounts of human IL8 (for example hamster CHO cells, murine NCTC cells or human HEK293 cells), were transfected with the method of choice with empty vector and an expression plasmid encoding the FcγR or TNFL of interest. Next day, successful transfection is controlled by flow cytometry and when adherent TNFR responder cells are used, their supernatant is replaced by fresh medium to minimize the background of constitutive IL8 production. Control and FcγR transfectants were then pairwise added to the TNFR responder cells (typically in a 1:1 ratio) and the two types of cocultures were supplemented with serial dilutions of the antibody of interest (**Figure 5A**). After an additional day, the coculture SNs are analyzed by ELISA for their IL8 content. We have yet no evidence that genetic tagging of antibodies and antibody fusion proteins as shown in **Figure 1** affects FcγR binding (20). The evaluation of the FcγR-dependent agonism as describe can be done with Ab-GpL-Fps but is, of course, identically performable with conventional “GpL-free” variants.

In principle, the assay described can also be performed with stable FcγR transfectants or cells with endogenous FcγR expression. In this case, one should keep in mind the FcγR expression levels. If the latter are rather low in comparison to the TNFR expression levels, the maximum achievable TNFR response might be dampened due to the limited number of FcγRs available, which only allows partial occupation of the TNFR pool by FcγR-bound antibodies. Cell culture supernatants are sufficient to perform the assay described. Two issues, however, must be kept in mind. First, when produced with the help of human cells, the antibody-containing supernatants may already contain significant amounts of IL8 released from the antibody producing transfectants. This TNFR engagement-independent IL8 background occurs in both EV and FcγR cocultures and can be falsely interpreted as intrinsic agonism of the antibody, especially when the antibody supernatants were used at low dilution. Thus, we recommend to analyze the SN used for stimulation of the TNFR responder cells also directly in the IL8 ELISA. Second, a similar misinterpretation can happen when the antibody supernatants contain unknown producer cell-derived factors able to induce IL8 production in the TNFR responder cells. This issue can be controlled by analysis of antibody-free control supernatants and/or the use of variants of the TNFR responder cells lacking TNFR expression.

Examples for this type of assay are shown in **Figures 5B, C**. Fc or Fc-GpL fusion proteins with N-terminal VHH domains specific for the category II TNFRs CD40 (VHH V12t, ref. 30), 41BB (VHH 4H04, ref. 31), and OX40 (VHH 1D10V1, ref. 36) were poorly active on TNFR responder cells in coculture with vector transfected cells but gain high activity in the presence of FcγR1A transfected cells (**Figure 5B**). It is worth mentioning that a VHH-Fc fusion protein targeting the category I TNFR GITR (VHH hzC06, ref. 37) already shows activity in the presence of vector transfected control cells and only moderately benefited from FcγR1A transfected cells (**Figure 5B**). Hexavalent VHH-Fc-GpL fusion protein variants were already highly active in the presence of EV transfected control cells and obviously act agonistic independent from FcγR binding (**Figure 5C**). These results resemble in this respect conventional bivalent IgG1 antibodies targeting these receptors (34).

Please be aware, anti-TNFR antibodies typically display FcγR-independent agonistic activity upon oligomerization with protein G or secondary antibodies (12). Therefore, TNFR-specific antibodies or antibody fusion proteins with high FcγR-independent activity have to be checked for aggregated antibody species and their specific activity compared to the non-aggregated molecule species. This can be done, for example, by preparative gel filtration and functional re-analysis of the differently aggregated protein species, if there are any.

## 5 Discussion

Advantages: The major advantage of the use of GpL fusion protein-based protocols for the characterization anti-TNFR antibodies is certainly their simple and broad applicability along with their straightforward implementability for cell biology and immunology laboratories without dedicated, and often quite expensive, biophysical instrumentation for quantitative analysis of protein-protein interactions. Eventually, antibody GpL fusion proteins are labeled antibodies, but without all the challenges associated with chemically or physically labeling procedures (need for purified proteins, heterogeneity of labeled antibodies, reproducibility of labeling reaction) or the need for secondary detection of a tag with all its potential pitfalls. Since the antibody GpL fusion proteins are “intrinsically” labeled, they combine the possibilities of cell-free methods using label-free proteins and cell-based methods requiring directly (chemical modification) or indirectly labeled antibodies (secondary antibodies anti-tag antibodies etc.) (**Table 2**). Furthermore, the GpL tag ensures high sensitivity and linear quantification over several orders of magnitude making it superior to ELISA or flow cytometry based protocols.

**Table 2:** Methods for the analysis of the interaction of TNFRs with anti-TNFR antibodies and anti-TNFR antibody fusion proteins.

Method	Purification Needed	Labeling needed	Immobilization required	Cell-based assays	Required equipment	Tracer Applications
Surface plasmon resonance (SRP)	Set-up Dependent	No	Yes	No	SPR system	No
Microscale thermophoresis (MST)	No	No	No	No	MST instrument	No
Biolayer interferometry (BLI)	Yes	No	Yes	No	Biolayer interferometer	No
Isothermal titration calorimetry (ITC)	No	No	No	No	Calorimeter	No
ELISA	Set-up-dependent	Yes	Yes	No	ELISA microplate reader	No
Flow cytometry	No	Yes	No	Yes	Flow cytometer	No
Cellular/cell-free GpL-αTNFR fusion proteins	No	No	yes (cell-free) no (cellular)	Yes	Luminescence microplate reader	Yes

Limitations and pitfalls: The cell-free and cell-based procedures described for the determination of antibody (or ligand) affinity for TNFRs can be straightforwardly used in time resolved manner for kinetic experiments aiming on the determination of association and dissociation rate constants and T_1/2_ of the antibody/ligand-TNFR complexes formed (23, 38). The binding study protocols with antibody GpL fusion proteins involve manual non-automatic steps for the removal of unbound molecules. These steps require still a few seconds even when adherent cells or immobilized proteins are used and the buffer in all wells is discarded in parallel by a rapid hand movement of the whole plate. The time needed for removal of unbound proteins is even longer when suspension cells are used. Thus, association and dissociation processes significantly occurring in this time frame or faster are not reliable resolvable. This limitation also accounts for ELISA or flow cytometry methods but is hardly relevant for automated biophysical methods, e.g. SPR and BLI. Indeed, the latter allow to study antibody-antigen interactions in real-time and can also be used to analyse transient binding events. As for any other enzyme, too, the activity of the GpL domain is dependent on temperature and buffer conditions. Therefore, it is crucial to ensure that all samples are similarly processed and washed so that the luciferase activity of all samples is finally measured under identical buffer conditions.

As already discussed at the beginning, a prerequisite for the binding studies protocols described is the knowledge of the amino acid or DNA sequence of the antibody or antibody variant of interest to allow the cloning of the *Gaussia princeps* luciferase (GpL) fusion protein needed. For the huge majority of preclinical or clinical relevant anti-TNFR antibodies this information is, however, straightforwardly available from corresponding patents. Although it appears plausible that tagging of the C-terminus of the heavy or light chain of an IgG with a stable monomeric protein domain as the GpL domain does not affect the antigen binding properties of the Fab domains of the antibody, this cannot been ruled out with absolute certainty. However, this issue can be experimentally controlled/verified by homotypic competition assays, in which the parental non-modified antibody is used as a competitor for its GpL-tagged counterpart. Lack of interference of the GpL domain with antigen binding in such experiments is then demonstrated when the K_i_-value obtained for the parental antibody does not significantly differ from the K_D_-value of the GpL-Ab-Fp. Due the excellent linearity of the luciferase read-out (23, 38) and as long as luciferase activity has been recorded for all samples of an experiment after the same time after addition of substrate, the major source of variation derives from the handling by the experimenter. In the recent years, we frequently used cellular equilibrium binding studies with GpL antibody fusion proteins to determine the antibody affinity for cell surface-exposed antigens or FcγRs (20–22). In these studies, the standard error of mean of the affinities obtained was typically below 50% when 4-6 independently performed binding experiments were analyzed.

## 6 Materials and methods

### 6.1 Reagents and cell lines

HEK293T, HT1080 (both ATCC, Rockville, USA), HT1080-CD40, HT1080-41BB, HT1080-GITR (all ref (39)., HT1080-OX40 (40) and HT1080-Bcl2-TNFR2 cells (41) were cultivated in RPMI 1640 medium (Sigma-Aldrich, Steinheim, Germany) supplemented with 10% fetal calf serum (FCS; GIBCO) under standard conditions (37°C, 5% CO_2_). To obtain expression plasmids encoding the various antibodies constructs, fusion proteins and ligands used, corresponding synthetic DNA fragments and PCR amplicons have been cloned in the expression vector pCR3 (Invitrogen, Germany). Amino acid sequences of the fusion proteins generated this way have been collected in **Supplemental Table S1**. Plasmids for production of antibodies are listed in **Tables S2** and **S3** gives reference to the sources of the amino acid sequences. Expression plasmids for FcγRI (CD64) (in pCMV-Sport6) was obtained from SourceBioScience (Nottingham, UK). The IL8 ELISA Kit from BD Biosciences (San Diego, USA) was used to determine human IL8 and OD values were measured with PHOmo photometer (anthos Mikrosysteme GmbH, Frieoythe, Germany). Protein G was obtained from Sigma-Aldrich (Steinheim, Germany). The GpL substrate coelenterazine was obtained from Carl Roth (Karlsruhe, Germany) and RLU were measured with LUmo luminometer (anthos Mikrosysteme GmbH, Frieoythe, Germany).

### 6.2 Expression of recombinant proteins

Recombinant proteins were expressed in HEK293T cells by transient transfection with the expression plasmids of interest using PEI (polyethylenimine; Polyscience Inc., Warrington, USA) as described elsewhere (42). Concentrations of the recombinant antibodies and antibody fusion proteins in cell culture supernatants were evaluated by western blot comparison with a purified antibody standard of known concentration essentially as described in ref. 42 for GpL-PGRN and purified rec. PGRN (42). In brief, a serial dilution of the standard protein and the antibody fusion protein-containing cell culture supernatant were processed in parallel on the same gel/blot and finally the concentration of the non-purified antibody variant in the supernatant was estimated according to the standard sample yielding similar band intensities. Alternatively, the concentration of a GpL-Ab-Fp in the supernatant can be determined using a commercially available IgG ELISA kit or by measuring its luciferase activity and comparison or a purified GpL fusion protein of known concentration.

### 6.3 Cellular equilibrium binding studies

For equilibrium binding studies with adherent cells, control cells without TNFR expression and cells stably or transiently transfected with the TNFR type of interest were seeded (1 x or 2 x 10^4^ per well, dependent on cell size) in black clear bottom 96-well cell culture plates overnight. Next day, control cells and TNFR expressing cells were pairwise incubated with a two-fold dilution series (9-11 sequential dilution steps) of the GpL antibody fusion protein (GpL-Ab-Fp) of interest at 37°C for 30 min to 1 h. A typically starting concentration for the GpL-Ab-Fp is 2 µg/ml. Cells/plates were then washed five times with icecold PBS to remove unbound GpL-Ab-Fp molecules. Finally, 50 µl RPMI 1640 medium supplemented with 0,5% FCS, 1% Pen/Strep (GpL medium) were added to each well. Luminescence was then measured as described below separately. The binding values derived of the TNFR expressing cells were considered as total binding and the binding values of the paired TNFR negative control cells were considered as non-specific binding. Specific binding was calculated by subtraction of unspecific binding values from the total binding values. K_D_ values were finally calculated by analyzing the specific binding values using the built-in “one site specific binding” function of the GraphPad Prism 5 software which fits the data to a function of the type Y = Bmax*X/(K_D_ + X) under minimization of the sum of squares (non-linear regression). Y = specific binding, Bmax = maximal specific binding, K_D_ = dissociation constant, and X = ligand/antibody concentration.

When no TNFR-negative control cells are available to determine non-specific binding two modifications of the procedure described are possible. In both cases, the same TNFR-positive cells used for determination of the total binding values were also seeded/used to obtain the non-specific binding values. A first protocol modification to determine non-specific binding of the GpL-Ab-Fp is then to preincubate the control cells for 1 h with an excess of the “GpL”-free version of the antibody (x200 of the starting concentration of GpL-Ab-Fp) before adding the GpL-Ab-Fp dilution series. A second possible protocol modification to determine non-specific binding of the GpL-Ab-Fp is to incubate the control cells instead with the GpL-Ab-Fp targeting the TNFR of interest with a structurally similar GpL-Ab-Fp with irrelevant specificity, thus recognizing a target not expressed on the cells used for the binding study.

For equilibrium binding studies with suspension or poorly adherent cells (e.g. HEK293 cells), cells were aliquoted in 1 ml of medium (0,1 – 1 x 10^6^ cells). Afterwards the cell aliquots were grouped and treated with the GpL-Ab-Fp as described above for adherent cells. For removal of unbound antibody molecules by washing with icecold PBS, the various cell samples were transferred to Eppendorf tubes and centrifuged 30 sec. at 14.000 rpm. The cell pellets were then resuspended in 1 ml icecold PBS and the washing procedure was repeated 3 times. After the last washing cycle, cells were resuspended in 50 µl RPMI 1640 medium supplemented with 0,5% FCS, 1% Pen/Strep, transferred to a black 96-well plate and further processed as described above for adherent cells.

### 6.4 Cell-free equilibrium binding studies

To perform cell free binding studies, black high binding 96-well plates (Greiner Bio-One) were incubated overnight at 4°C with 1 µg/ml of the recombinant protein of interest in coating buffer (0,1 M carbonate buffer). For the determination of unspecific binding, 1 µg/ml of a corresponding irrelevant TNFR fusion protein in coating buffer were used for coating. Next day, after three washing steps with PBST, plates were blocked one hour with 10% FCS in PBS. After additional three washing steps with PBST, the differently coated wells were pairwise incubated with a two-fold dilution series (9-11 sequential dilution steps) of the GpL antibody fusion protein (GpL-Ab-Fp) of interest at 37°C for 1 h. A typically starting concentration for the GpL-Ab-Fp is 2 µg/ml. Plates were then further processed as described above in the protocol for adherent cells. In the case that no irrelevant recombinant TNFR protein is available to determine non-specific binding, similar modifications of the procedure are possible as described above for the cellular binding studies. In the first modified protocol, wells coated with the recombinant TNFR protein of interest and pretreated with an excess of the GpL”-free version of the corresponding GpL-Ab-Fp were processed to obtain non-specific binding values. In the second modified protocol, wells coated with the recombinant TNFR protein of interest and incubated with a dilution series of an irrIgG1-LC : GpL fusion protein instead with a dilution series of the TNFR-specific GpL-Ab-Fp were analyzed to obtain non-specific binding values.

### 6.5 Binding domain/epitope mapping by cell-free binding studies

For this application black high binding 96-well plates (Greiner Bio-One) were coated with protein G (Millipore; 1 µg/ml in 0,1 M carbonate buffer) overnight at 4°C. Next day, after three washing steps with PBST, plates were blocked one hour with 10% FCS in PBS. After additional three wash cycles, the anti-TNFR antibody of interest and a corresponding control antibody of irrelevant specificity (1 µg/ml) were added in medium for 1 h at 37°C. After removal of unbound antibody molecules by three washing steps with PBST, GpL-tagged deletion mutants of the ectodomain of the TNFR of interest (500 ng/ml) were added in medium at 37°C for two hours. Plates were then processed as described above and specific binding values were calculated by subtraction of the non-specific binding values obtained with irrelevant control antibody from the total binding values derived of the samples with the anti-TNF of interest. Lack of specific binding of a particular GpL-tagged TNFR deletion mutant indicated then that the domain/epitope recognized by the antibody is destroyed or missing in this mutant.

### 6.6 Determination of luminescence activity

To quantify the cell-associated and/or well-associated GpL-Ab-Fp activity after the last washing steps of a binding study, 50 µl RPMI 1640 medium supplemented with 0,5% FCS, 1% Pen/Strep (GpL medium) were added to each well. To start the luminescence reaction, 25 µl of GpL substrate solution (coelenterazine 1,5 µM in PBS; Carl Roth, Karlsruhe, Germany) were added and luminescence was immediately measured with a luminometer (LuMo anthos Mikrosysteme GmbH, Friesoythe, Germany).

### 6.7 Evaluation of FcγR-dependent TNFR agonism

To evaluate the intrinsic and FcγR-dependent activity of antibodies targeting TNFRs their ability to stimulate IL8 production was determined. Therefore, HT1080-transfectants stably expressing the TNFR of interest were seeded in 96-well plates (2 x 10^4^ per well). In parallel, HEK293 cells were transiently transfected with empty vector (EV) or an expression plasmid encoding the FcγR of interest (in the example shown in **Figure 5** FcγRI (CD64)). The next day, the medium of the HT1080-TNFR transfectants was replaced by fresh medium to minimize the background of the constitutive IL8 production. HT1080-TNFR cells were then supplemented with EV- or FcγR-transfected HEK293 cells (2 x 10^4^ per well). HT1080-TNFR cocultures with HEK293-EV and HEK293-FcγR transfectants were then pairwise stimulated with a two-fold dilution series (9 sequential dilution steps) of the antibody variant of interest overnight. Finally, the coculture supernatants were analyzed for their IL8 content using the BD OptEIA™ human IL8-ELISA kit.

## Data availability statement

The original contributions presented in the study are included in the article/**Supplementary Material**. Further inquiries can be directed to the corresponding author.

## Author contributions

OZ, MA, and IL performed experiments. HW and IL wrote the manuscript. All authors contributed to the article and approved the submitted version.
