# Supplementary material for: Basic characterization of antibodies targeting receptors of the tumor necrosis factor receptor superfamily
Source: Front Immunol. 2023 Mar 27;14:1115667. doi: 10.3389/fimmu.2023.1115667 (PMC10083269; doi:10.3389/fimmu.2023.1115667)
Supplement: Supplementary file 1 [file DataSheet_1.pdf]

Supplemental Data

Supplemental Table S1. pCR3-based expression plasmids and corresponding amino acid sequences.

Leader: underlined; Flag tag: underlined + grey background; restriction site encoding 2AA linker: bold; linker: bold + italic; variable domains: italic; TNC trimerization domain: italic + underlined + grey background; IgG1(N297A), Fc, Fc(DANA): grey background; TNFR domains: bold + grey background

1	anti-CD40(G28.5)-Flag-HC-IgG1-pCR3	MNFGFSLIFLVLVLKGVQCEVKLVPRQLDYKDDDDKELDIQLQQSGPGLVKPSQSLSLTCS<br>VTGYSITTNYNWNWIRQFPGNKLEWMGYIRYDGTSEYTPSLKNRVSITRDTSMNQFFLRLT<br>SVTPEDTATYYCARLDYWGQGTLVTVSSGSSSASTKGPSVFPLAPSSKSTSGGTAALGCLV<br>KDYFPEPVTVSWNSGALTSGVHTFPAVLQSSGLYSLSSVVTVPSSSLGTQTYICNVNHKPS<br>NTKVDKKVEPKSCDKTHTCPPCPAPELLGGPSVFLFPPKPKDTLMISRTPEVTCVVVDVSH<br>EDPEVKFNWYVDGVEVHNAKTKPREEQYNSTYRVVSVLTVLHQDWLNGKEYKCKVSNKALP<br>APIEKTISKAKGQPREPQVYTLPPSRDELTKNQVSLTCLVKGFYPSDIAVEWESNGQPENN<br>YKTTPPVLDSDGSFFLYSKLTVDKSRWQQGNVFSCSVMHEALHNHYTQKSLSLSPGK
2	anti-CD40(G28.5)-Flag-LC-pCR3	MNFGFSLIFLVLVLKGVQCEVKLVPRQLDYKDDDDKELDIVMTQNPLSLPVSLGDEASISC<br>RSSQSLENSNGNTFLNWFFQKPGQSPQLLIYRVSNRFSGVPDRFSGSGSGTDFTLKISRVE<br>AEDLGVYFCLQVTHVPYTFGGGTTLEIKGSEIKRTVAAPSVFIFPPSDEQLKSGTASVVCL<br>LNNFYPREAKVQWKVDNALQSGNSQESVTEQDSKDSTYSLSSTLTLSKADYEKHKVYACEV<br>THQGLSSPVTKSFNRGEC
3	anti-CD40(G28.5)-Flag-LC:GpL-pCR3	MNFGFSLIFLVLVLKGVQCEVKLVPRQLDYKDDDDKELDIVMTQNPLSLPVSLGDEASISC<br>RSSQSLENSNGNTFLNWFFQKPGQSPQLLIYRVSNRFSGVPDRFSGSGSGTDFTLKISRVE<br>AEDLGVYFCLQVTHVPYTFGGGTTLEIKGSEIKRTVAAPSVFIFPPSDEQLKSGTASVVCL<br>LNNFYPREAKVQWKVDNALQSGNSQESVTEQDSKDSTYSLSSTLTLSKADYEKHKVYACEV<br>THQGLSSPVTKSFNRGECLEKPTENNEDFNIVAVASNFATTDLDADRGKLPGKKLPLEVLK<br>EMEANARKAGCTRGCLICLSHIKCTPKMKKFIPGRCHTYEGDKESAQGGIGEAIVDIPEIP<br>GFKDLEPMEQFIAQVDLCVDCTTGCLKGLANVQCSDLLKKWLPQRCATFASKIQGQVDKIK<br>GAGGD
4	TNFR2(CRD1-4-stalk)(=ed)(h)-Flag-GpL-pCR3	MAPVAVWAALAVGLELWAAAHALPAQVAFTPYAPEPGSTCRLREYYDQTAQMCCSKCSPGQ<br>HAKVFCTKTSDTVCDSCEDSTYTQLWNWVPECLSCGSRCSSDQVETQACTREQNRICTCRP<br>GWYCALSKQEGCRLCAPLRKCRPGFGVARPGTETSDVVCKPCAPGTFSNTTSSTDICRPHQ<br>ICNVVAIPGNASMDAVCTSTSPTRSMAPGAVHLPQPVSTRSQHTQPTPEPSTAPSTSFLLP<br>MGPSPPAEGSTGDGSAGEFDYKDDDDKLEKPTENNEDFNIVAVASNFATTDLDADRGKLPG<br>KKLPLEVLKEMEANARKAGCTRGCLICLSHIKCTPKMKKFIPGRCHTYEGDKESAQGGIGE<br>AIVDIPEIPGFKDLEPMEQFIAQVDLCVDCTTGCLKGLANVQCSDLLKKWLPQRCATFASK<br>IQGQVDKIKGAGGD
5	TNFR2(CRD1-4)(=Δstalk)(h)-2xFlag-GpL-pCR3	MAPVAVWAALAVGLELWAAAHALPAQVAFTPYAPEPGSTCRLREYYDQTAQMCCSKCSPGQ<br>HAKVFCTKTSDTVCDSCEDSTYTQLWNWVPECLSCGSRCSSDQVETQACTREQNRICTCRP<br>GWYCALSKQEGCRLCAPLRKCRPGFGVARPGTETSDVVCKPCAPGTFSNTTSSTDICRPHQ<br>ICNVVAIPGNASMDAVCTGSDYKDDDDKEFDYKDDDDKLEKPTENNEDFNIVAVASNFATT<br>DLDADRGKLPGKKLPLEVLKEMEANARKAGCTRGCLICLSHIKCTPKMKKFIPGRCHTYEG<br>DKESAQGGIGEAIVDIPEIPGFKDLEPMEQFIAQVDLCVDCTTGCLKGLANVQCSDLLKKW<br>LPQRCATFASKIQGQVDKIKGAGGD
6	TNFR2(CRD1,2,3-loop)(h)-Flag-GpL-pCR3	MAPVAVWAALAVGLELWAAAHALPAQVAFTPYAPEPGSTCRLREYYDQTAQMCCSKCSPGQ<br>HAKVFCTKTSDTVCDSCEDSTYTQLWNWVPECLSCGSRCSSDQVETQACTREQNRICTCRP<br>GWYCALSKQEGCRLCAPLRKCRPGFGVARPGTETSDVVGSAGEFDYKDDDDKLEKPTENNE<br>DFNIVAVASNFATTDLDADRGKLPGKKLPLEVLKEMEANARKAGCTRGCLICLSHIKCTPK<br>MKKFIPGRCHTYEGDKESAQGGIGEAIVDIPEIPGFKDLEPMEQFIAQVDLCVDCTTGCLK<br>GLANVQCSDLLKKWLPQRCATFASKIQGQVDKIKGAGGD
7	TNFR2(CRD1,2)(h)-Flag-GpL-pCR3	MAPVAVWAALAVGLELWAAAHALPAQVAFTPYAPEPGSTCRLREYYDQTAQMCCSKCSPGQ<br>HAKVFCTKTSDTVCDSCEDSTYTQLWNWVPECLSCGSRCSSDQVETQACTREQNRICTGSA<br>GEFDYKDDDDKLEKPTENNEDFNIVAVASNFATTDLDADRGKLPGKKLPLEVLKEMEANAR<br>KAGCTRGCLICLSHIKCTPKMKKFIPGRCHTYEGDKESAQGGIGEAIVDIPEIPGFKDLEP<br>MEQFIAQVDLCVDCTTGCLKGLANVQCSDLLKKWLPQRCATFASKIQGQVDKIKGAGGD
8	TNFR2(CRD1)(=PLAD)(h)-2xFlag-GpL-pCR3	MAPVAVWAALAVGLELWAAAHALPAQVAFTPYAPEPGSTCRLREYYDQTAQMCCSKCSPGQ<br>HAKVFCTKTSDTVCDGSDYKDDDDKEFDYKDDDDKLEKPTENNEDFNIVAVASNFATTDLD<br>ADRGKLPGKKLPLEVLKEMEANARKAGCTRGCLICLSHIKCTPKMKKFIPGRCHTYEGDKE<br>SAQGGIGEAIVDIPEIPGFKDLEPMEQFIAQVDLCVDCTTGCLKGLANVQCSDLLKKWLPQ<br>RCATFASKIQGQVDKIKGAGGD
9	anti-TNFR2(68/69)-Flag-HC-N297A-pCR3	MNFGFSLIFLVLVLKGVQCEVKLVPRQLDYKDDDDKEFQVTLKESGPALVKPTQTLTLTCT<br>FSGFSLSTSGMGVGWIRQPPGKALEWLAHIWWDDDKFYNPSLKSRLTISKDTSKNQVVLTM<br>TNMDPVDTATYYCARLTGTRYFDYWGQGTTVTVSSRSSSASTKGPSVFPLAPSSKSTSGGT<br>AALGCLVKDYFPEPVTVSWNSGALTSGVHTFPAVLQSSGLYSLSSVVTVPSSSLGTQTYIC<br>NVNHKPSNTKVDKKVEPKSCDKTHTCPPCPAPELLGGPSVFLFPPKPKDTLMISRTPEVTC<br>VVVDVSHEDPEVKFNWYVDGVEVHNAKTKPREEQYASTYRVVSVLTVLHQDWLNGKEYKCK<br>VSNKALPAPIEKTISKAKGQPREPQVYTLPPSRDELTKNQVSLTCLVKGFYPSDIAVEWES<br>NGQPENNYKTTPPVLDSDGSFFLYSKLTVDKSRWQQGNVFSCSVMHEALHNHYTQKSLSLS<br>PGK
10	anti-TNFR2(68/69)-Flag-	MNFGFSLIFLVLVLKGVQCEVKLVPRQLDYKDDDDKELDVQMTQSPSSLSASVGDRVTITC<br>KASQDINKFIAWYQQKPGKAPKLLIHYTSTLQPGIPSRFSGSGSGRDYTFTISSLQPEDIA

	LC-pCR3	TYYCLQYGNLWTFGGGTKVEIKGSEIKRTVAAPSVFIFPPSDEQLKSGTASVVCLLNNFYP<br>REAKVQWKVDNALQSGNSQESVTEQDSKDSTYSLSSTLTLSKADYEKHKVYACEVTHQGLS<br>SPVTKSFNRGEC
11	anti-CD40(CP-8…)-Flag-HC-IgG1-pCR3	MNFGFSLIFLVLVLKGVQCEVKLVPRQLDYKDDDDKEFQVQLVQSGAEVKKPGASVKVSCK<br>ASGYTFTGYYMHWVRQAPGQGLEWMGWINPDSGGTNYAQKFQGRVTMTRDTSISTAYMELN<br>RLRSDDTAVYYCARDQPLGYCTNGVCSYFDYWGQGTLVTVSSRSSSASTKGPSVFPLAPSS<br>KSTSGGTAALGCLVKDYFPEPVTVSWNSGALTSGVHTFPAVLQSSGLYSLSSVVTVPSSSL<br>GTQTYICNVNHKPSNTKVDKKVEPKSCDKTHTCPPCPAPELLGGPSVFLFPPKPKDTLMIS<br>RTPEVTCVVVDVSHEDPEVKFNWYVDGVEVHNAKTKPREEQYNSTYRVVSVLTVLHQDWLN<br>GKEYKCKVSNKALPAPIEKTISKAKGQPREPQVYTLPPSRDELTKNQVSLTCLVKGFYPSD<br>IAVEWESNGQPENNYKTTPPVLDSDGSFFLYSKLTVDKSRWQQGNVFSCSVMHEALHNHYT<br>QKSLSLSPGK
12	anti-CD40(CP-8…)-Flag-LC-pCR3	MNFGFSLIFLVLVLKGVQCEVKLVPRQLDYKDDDDKELDIQMTQSPSSVSASVGDRVTITC<br>RASQGIYSWLAWYQQKPGKAPNLLIYTASTLQSGVPSRFSGSGSGTDFTLTISSLQPEDFA<br>TYYCQQANIFPLTFGGGTKVEIKGSEIKRTVAAPSVFIFPPSDEQLKSGTASVVCLLNNFY<br>PREAKVQWKVDNALQSGNSQESVTEQDSKDSTYSLSSTLTLSKADYEKHKVYACEVTHQGL<br>SSPVTKSFNRGEC
13	anti-CD40(ADC)-Flag-HC-IgG1-pCR3	MNFGFSLIFLVLVLKGVQCEVKLVPRQLDYKDDDDKEFEVQLLESGGGLVQPGGSLRLSCA<br>ASGFTFSTYGMHWVRQAPGKGLEWLSYISGGSSYIFYADSVRGRFTISRDNSENALYLQMN<br>SLRAEDTAVYYCARILRGGSGMDLWGQGTLVTVSSRSSSASTKGPSVFPLAPSSKSTSGGT<br>AALGCLVKDYFPEPVTVSWNSGALTSGVHTFPAVLQSSGLYSLSSVVTVPSSSLGTQTYIC<br>NVNHKPSNTKVDKKVEPKSCDKTHTCPPCPAPELLGGPSVFLFPPKPKDTLMISRTPEVTC<br>VVVDVSHEDPEVKFNWYVDGVEVHNAKTKPREEQYNSTYRVVSVLTVLHQDWLNGKEYKCK<br>VSNKALPAPIEKTISKAKGQPREPQVYTLPPSRDELTKNQVSLTCLVKGFYPSDIAVEWES<br>NGQPENNYKTTPPVLDSDGSFFLYSKLTVDKSRWQQGNVFSCSVMHEALHNHYTQKSLSLS<br>PGK
14	anti-CD40(ADC)-Flag-LC-pCR3	MNFGFSLIFLVLVLKGVQCEVKLVPRQLDYKDDDDKELQSVLTQPPSASGTPGQRVTISCT<br>GSSSNIGAGYNVYWYQQLPGTAPKLLIYGNINRPSGVPDRFSGSKSGTSASLAISGLRSED<br>EADYYCAAWDKSISGLVFGGGTKLTVLGGSEIKRTVAAPSVFIFPPSDEQLKSGTASVVCL<br>LNNFYPREAKVQWKVDNALQSGNSQESVTEQDSKDSTYSLSSTLTLSKADYEKHKVYACEV<br>THQGLSSPVTKSFNRGEC
15	3xVHH(OX40(1D10V1))-Fc(DANA)-Flag-pCR3	MNFGFSLIFLVLVLKGVQCEVKLVPRGTEVQLLESGGGEVQPGGSLRLSCAASGFTFSDAF<br>MYWVRQAPGKGLEWVSSISNRGLKTAYAESVKGRFTISRDNAKNTLYLQMSSLRAEDTAVY<br>YCSRDVDGDFRGQGTLVTVKPRSGGGGSGGGGSGGGGSGGGGSGGGGSEVQLLESGGGEVQ<br>PGGSLRLSCAASGFTFSDAFMYWVRQAPGKGLEWVSSISNRGLKTAYAESVKGRFTISRDN<br>AKNTLYLQMSSLRAEDTAVYYCSRDVDGDFRGQGTLVTVKPGGGGSGGGGSGGGGSGGGSS<br>GGGGSQLEVQLLESGGGEVQPGGSLRLSCAASGFTFSDAFMYWVRQAPGKGLEWVSSISNR<br>GLKTAYAESVKGRFTISRDNAKNTLYLQMSSLRAEDTAVYYCSRDVDGDFRGQGTLVTVKP<br>GSKTHTCPPCPAPELLGGPSVFLFPPKPKDTLMISRTPEVTCVVVAVSHEDPEVKFNWYVD<br>GVEVHNAKTKPREEQYASTYRVVSVLTVLHQDWLNGKEYKCKVSNKALPAPIEKTISKAKG<br>QPREPQVYTLPPSRDELTKNQVSLTCLVKGFYPSDIAVEWESNGQPENNYKTTPPVLDSDG<br>SFFLYSKLTVDKSRWQQGNVFSCSVMHEALHNHYTQKSLSLSPGKEFDYKDDDDKLE
16	3xVHH(41BB(4H04))-Fc(DANA)-Flag-pCR3	MNFGFSLIFLVLVLKGVQCEVKLVPRGTQVQLVESGGGVVQPGRSLRLSCAASGSTFSIVA<br>MGWYRQAPGKQRELVASIITGDGDTNYADSVKGRFTISRDNSKNTMYLQMNSLKPEDTAVY<br>YCYARTGYGSSWLMGHEYDYWGQGTQVTVSSLGRSGGGGSGGGGSGGGGSGGGGSGGGGSQ<br>VQLVESGGGVVQPGRSLRLSCAASGSTFSIVAMGWYRQAPGKQRELVASIITGDGDTNYAD<br>SVKGRFTISRDNSKNTMYLQMNSLKPEDTAVYYCYARTGYGSSWLMGHEYDYWGQGTQVTV<br>SSLGGGGGSGGGGSGGGGSGGGGSGGGGSQLQVQLVESGGGVVQPGRSLRLSCAASGSTFS<br>IVAMGWYRQAPGKQRELVASIITGDGDTNYADSVKGRFTISRDNSKNTMYLQMNSLKPEDT<br>AVYYCYARTGYGSSWLMGHEYDYWGQGTQVTVSSLGGSKTHTCPPCPAPELLGGPSVFLFP<br>PKPKDTLMISRTPEVTCVVVAVSHEDPEVKFNWYVDGVEVHNAKTKPREEQYASTYRVVSV<br>LTVLHQDWLNGKEYKCKVSNKALPAPIEKTISKAKGQPREPQVYTLPPSRDELTKNQVSLT<br>CLVKGFYPSDIAVEWESNGQPENNYKTTPPVLDSDGSFFLYSKLTVDKSRWQQGNVFSCSV<br>MHEALHNHYTQKSLSLSPGKEFDYKDDDDKLE
17	VHH(CD40(V12t))-Fc-Flag-pCR3	MNFGFSLIFLVLVLKGVQCEVKLVPRQLQVQLQESGGGLVQAGGSLRLSCAASGLVFKRYS<br>MNWYRQPPGQQRGLVASISDSGVSTNYADSVKGRFTISRDNAKNIGYLQMNSLKPEDTAVY<br>YCNMHTFWGQGTQVTVSSGSKTHTCPPCPAPELLGGPSVFLFPPKPKDTLMISRTPEVTCV<br>VVDVSHEDPEVKFNWYVDGVEVHNAKTKPREEQYNSTYRVVSVLTVLHQDWLNGKEYKCKV<br>SNKALPAPIEKTISKAKGQPREPQVYTLPPSRDELTKNQVSLTCLVKGFYPSDIAVEWESN<br>GQPENNYKTTPPVLDSDGSFFLYSKLTVDKSRWQQGNVFSCSVMHEALHNHYTQKSLSLSP<br>GKEFDYKDDDDKLE
18	VHH(41BB(4H04))-Fc-Flag-GpL-pCR3	MNFGFSLIFLVLVLKGVQCEVKLVPRQLQVQLVESGGGVVQPGRSLRLSCAASGSTFSIVA<br>MGWYRQAPGKQRELVASIITGDGDTNYADSVKGRFTISRDNSKNTMYLQMNSLKPEDTAVY<br>YCYARTGYGSSWLMGHEYDYWGQGTQVTVSSLGGSKTHTCPPCPAPELLGGPSVFLFPPKP<br>KDTLMISRTPEVTCVVVDVSHEDPEVKFNWYVDGVEVHNAKTKPREEQYNSTYRVVSVLTV<br>LHQDWLNGKEYKCKVSNKALPAPIEKTISKAKGQPREPQVYTLPPSRDELTKNQVSLTCLV<br>KGFYPSDIAVEWESNGQPENNYKTTPPVLDSDGSFFLYSKLTVDKSRWQQGNVFSCSVMHE<br>ALHNHYTQKSLSLSPGKEFDYKDDDDKLEKPTENNEDFNIVAVASNFATTDLDADRGKLPG<br>KKLPLEVLKEMEANARKAGCTRGCLICLSHIKCTPKMKKFIPGRCHTYEGDKESAQGGIGE<br>AIVDIPEIPGFKDLEPMEQFIAQVDLCVDCTTGCLKGLANVQCSDLLKKWLPQRCATFASK<br>IQGQVDKIKGAGGD
19	VHH(OX40(1D10V1))-Fc-Flag-GpL-pCR3	MNFGFSLIFLVLVLKGVQCEVKLVPRQLEVQLLESGGGEVQPGGSLRLSCAASGFTFSDAF<br>MYWVRQAPGKGLEWVSSISNRGLKTAYAESVKGRFTISRDNAKNTLYLQMSSLRAEDTAVY<br>YCSRDVDGDFRGQGTLVTVKPGSKTHTCPPCPAPELLGGPSVFLFPPKPKDTLMISRTPEV<br>TCVVVDVSHEDPEVKFNWYVDGVEVHNAKTKPREEQYNSTYRVVSVLTVLHQDWLNGKEYK<br>CKVSNKALPAPIEKTISKAKGQPREPQVYTLPPSRDELTKNQVSLTCLVKGFYPSDIAVEW<br>ESNGQPENNYKTTPPVLDSDGSFFLYSKLTVDKSRWQQGNVFSCSVMHEALHNHYTQKSLS<br>LSPGKEFDYKDDDDKLEKPTENNEDFNIVAVASNFATTDLDADRGKLPGKKLPLEVLKEME

		ANARKAGCTRGCLICLSHIKCTPKMKKFIPGRCHTYEGDKESAQGGIGEAIVDIPEIPGFK<br>DLEPMEQFIAQVDLCVDCTTGCLKGLANVQCSDLLKKWLPQRCATFASKIQGQVDKIKGAG<br>GD
20	VHH(CD40((V12t))-Fc(DANA)-Flag-pCR3	MNFGFSLIFLVLVLKGVQCEVKLVPRQLQVQLQESGGGLVQAGGSLRLSCAASGLVFKRYS<br>MNWYRQPPGQQRGLVASISDSGVSTNYADSVKGRFTISRDNAKNIGYLQMNSLKPEDTAVY<br>YCNMHTFWGQGTQVTVSSGSKTHTCPPCPAPELLGGPSVFLFPPKPKDTLMISRTPEVTCV<br>VVAVSHEDPEVKFNWYVDGVEVHNAKTKPREEQYASTYRVVSVLTVLHQDWLNGKEYKCKV<br>SNKALPAPIEKTISKAKGQPREPQVYTLPPSRDELTKNQVSLTCLVKGFYPSDIAVEWESN<br>GQPENNYKTTPPVLDSDGSFFLYSKLTVDKSRWQQGNVFSCSVMHEALHNHYTQKSLSLSP<br>GKEFDYKDDDDKLE
21	3xVHH(CD40(V12t))-Fc(DANA)-Flag-pCR3	MNFGFSLIFLVLVLKGVQCEVKLVPRGTQVQLQESGGGLVQAGGSLRLSCAASGLVFKRYS<br>MNWYRQPPGQQRGLVASISDSGVSTNYADSVKGRFTISRDNAKNIGYLQMNSLKPEDTAVY<br>YCNMHTFWGQGTQVTVSSRSGGGGSGGGGSGGGGSGGGGSGGGGSQVQLQESGGGLVQAGG<br>SLRLSCAASGLVFKRYSMNWYRQPPGQQRGLVASISDSGVSTNYADSVKGRFTISRDNAKN<br>IGYLQMNSLKPEDTAVYYCNMHTFWGQGTQVTVSSGGGGSGGGGSGGGGSGGGGSGGGGSQ<br>LQVQLQESGGGLVQAGGSLRLSCAASGLVFKRYSMNWYRQPPGQQRGLVASISDSGVSTNY<br>ADSVKGRFTISRDNAKNIGYLQMNSLKPEDTAVYYCNMHTFWGQGTQVTVSSGSKTHTCPP<br>CPAPELLGGPSVFLFPPKPKDTLMISRTPEVTCVVVAVSHEDPEVKFNWYVDGVEVHNAKT<br>KPREEQYASTYRVVSVLTVLHQDWLNGKEYKCKVSNKALPAPIEKTISKAKGQPREPQVYT<br>LPPSRDELTKNQVSLTCLVKGFYPSDIAVEWESNGQPENNYKTTPPVLDSDGSFFLYSKLT<br>VDKSRWQQGNVFSCSVMHEALHNHYTQKSLSLSPGKEFDYKDDDDKLE
22	VHH(GITR(hzC06)-Fc-Flag-GpL-pCR3	MNFGFSLIFLVLVLKGVQCEVKLVPRQLEVQLLESGGGEVQPGGSLRLSCAASGSVFSIDA<br>MGWYRQAPGKGLELVSALSGISSATYAESVKGRFTISRDNAKNTLYLQMSSLRAEDTAVYY<br>CYADVSTGWGRDAHGYWGQGTLVTVGSKTHTCPPCPAPELLGGPSVFLFPPKPKDTLMISR<br>TPEVTCVVVDVSHEDPEVKFNWYVDGVEVHNAKTKPREEQYNSTYRVVSVLTVLHQDWLNG<br>KEYKCKVSNKALPAPIEKTISKAKGQPREPQVYTLPPSRDELTKNQVSLTCLVKGFYPSDI<br>AVEWESNGQPENNYKTTPPVLDSDGSFFLYSKLTVDKSRWQQGNVFSCSVMHEALHNHYTQ<br>KSLSLSPGKEFDYKDDDDKLEKPTENNEDFNIVAVASNFATTDLDADRGKLPGKKLPLEVL<br>KEMEANARKAGCTRGCLICLSHIKCTPKMKKFIPGRCHTYEGDKESAQGGIGEAIVDIPEI<br>PGFKDLEPMEQFIAQVDLCVDCTTGCLKGLANVQCSDLLKKWLPQRCATFASKIQGQVDKI<br>KGAGGD
23	3xVHH(GITR(hzC06))-Fc-Flag-GpL-pCR3	MNFGFSLIFLVLVLKGVQCEVKLVPRGTEVQLLESGGGEVQPGGSLRLSCAASGSVFSIDA<br>MGWYRQAPGKGLELVSALSGISSATYAESVKGRFTISRDNAKNTLYLQMSSLRAEDTAVYY<br>CYADVSTGWGRDAHGYWGQGTLVTVRSGGGGSGGGGSGGGGSGGGGSGGGGSEVQLLESGG<br>GEVQPGGSLRLSCAASGSVFSIDAMGWYRQAPGKGLELVSALSGISSATYAESVKGRFTIS<br>RDNAKNTLYLQMSSLRAEDTAVYYCYADVSTGWGRDAHGYWGQGTLVTVGGGGSGGGGSGG<br>GGSGGGGSGGGGSQLEVQLLESGGGEVQPGGSLRLSCAASGSVFSIDAMGWYRQAPGKGLE<br>LVSALSGISSATYAESVKGRFTISRDNAKNTLYLQMSSLRAEDTAVYYCYADVSTGWGRDA<br>HGYWGQGTLVTVGSKTHTCPPCPAPELLGGPSVFLFPPKPKDTLMISRTPEVTCVVVDVSH<br>EDPEVKFNWYVDGVEVHNAKTKPREEQYNSTYRVVSVLTVLHQDWLNGKEYKCKVSNKALP<br>APIEKTISKAKGQPREPQVYTLPPSRDELTKNQVSLTCLVKGFYPSDIAVEWESNGQPENN<br>YKTTPPVLDSDGSFFLYSKLTVDKSRWQQGNVFSCSVMHEALHNHYTQKSLSLSPGKEFDY<br>KDDDDKLEKPTENNEDFNIVAVASNFATTDLDADRGKLPGKKLPLEVLKEMEANARKAGCT<br>RGCLICLSHIKCTPKMKKFIPGRCHTYEGDKESAQGGIGEAIVDIPEIPGFKDLEPMEQFI<br>AQVDLCVDCTTGCLKGLANVQCSDLLKKWLPQRCATFASKIQGQVDKIKGAGGD

Supplemental Table S2. Plasmids used (see supplemental table S1) for production of antibody variants.

Proteine name	Plasmids(s)
αCD40(G28.5)-IgG1	1+2
αCD40(G28.5)-IgG1-LC:GpL	1+3
αTNFR2(68/69)-IgG1(N297A)	9+10
αCD40(ADC)-IgG1	13+14
αCD40(CP-8…)-IgG1	11+12
3xVHH(1D10V1)-Fc(DANA)	15
3xVHH(4H04)-Fc(DANA)	16
VHH(V12t)-Fc	17
VHH(4H04)-Fc-GpL	18
VHH(1D10V1)-Fc-GpL	19
VHH(V12t)-Fc(DANA)-	20
3xVHH(V12t)-Fc(DANA)-	21
VHH(hzC06)-Fc-GpL	22
3xVHH(hzC06)-Fc-GpL	23

Supplemental Table S3. Sources of amino acid sequences.

**Protein domain**	**Amino acids (aa) sequence and/or source**
Signal peptide	MNFGFSLIFLVLVLKGVQCEVKLVPR
Flag tag	DYKDDDDK
Tenascin C trimerization domain (TNC)	aa 110-139 of gene bank ac.: AAA49086.1
Restriction site encoded 2 aa linkers	QL encoded by Mfe1 (CAATTG)<br>EL encoded by EcoR1/Mfe1 (GAATTG)<br>GS encoded by BamH1 (GGATCC)<br>EF encoded by EcoR1 (GAATTC)<br>LE encoded by Xho1 (CTCGAG)
Gaussia princeps luciferase (GpL)	aa 18-185 of gene bank ac.: AAG54095
Constant heavy chain of human IgG1	aa 145-476 of gene bank ac.: AAA02914.1
Constant heavy chain of human IgG1(N297A)	aa 145-476 with N287A mutation of gene bank ac.: AAA02914.1
Constant light chain	aa 105-214 of gene bank ac.: BAA97671.1
Linker	GGGGSGGGGSGGGGSGGGGSGGGGS
TNFR2(ed)	aa 1-258 of gene bank ac.: AAA36755.1
TNFR2(ed)-CRD1-4	aa 1-201 of gene bank ac.: AAA36755.1
TNFR2(ed)-CRD1-3	aa 1-160 of gene bank ac.: AAA36755.1
TNFR2(ed)-CRD1-2	aa 1-119 of gene bank ac.: AAA36755.1
TNFR2(ed)-CDR1	aa 1-76 of gene bank ac.: AAA36755.1
CD40L	aa 116-261 of gene bank ac.: AAH71754.1
OX40L	aa 52-183 of gene bank ac.: NP_003317
41BBL	aa 85-254 of gene bank ac.: NP_003802
TNF	aa 85-233 of gene bank ac.: NP_000585
VH and VL of G28.5 domain	aa 148-259 (VL) and aa 21-130 (HC) from scFv gene bank ac: sequence) AJ853736
Selicrelumab (CP8-…)	aa 1-117 (VL), aa 1-126 (VH) from KEGG drug database entry D11491
Mitazalimab (ADC)	aa 1-110 (VL), aa 1-119 (VH) from Thera-SAbDab[1]
VHH V12t (CD40-specific)	De Weerdt et al., 2021 ref. 30
VL and VH of 68/69 TNFR2	US patent US10988543B2
VHH OX40(1D10V1)	AU patent AU2019321490A1
VHH 41BB (4H04)	US patent US10501551B2
VHH GITR (hzC06)	US patent US2019100594A1

[1] Therapeutic Structural Antibody Database
